# Readability Optimization of Layperson Summaries in Urological Oncology Clinical Trials: Outcomes from the BRIDGE-AI 8 Study

**DOI:** 10.3390/curroncol32120696

**Published:** 2025-12-10

**Authors:** Ilicia Cano, Aalamnoor Pannu, Ethan Layne, Conner Ganjavi, Aditya Desai, Gus Miranda, Jie Cai, Vasileios Magoulianitis, Karan Gill, Gerhard Fuchs, Mihir Desai, Inderbir Gill, Giovanni E. Cacciamani

**Affiliations:** 1 USC Institute of Urology, Catherine and Joseph Aresty Department of Urology, Keck School of Medicine, University of Southern California, Los Angeles, CA 90089, USA; ilicia.cano@med.usc.edu (I.C.); aalamnoor.pannu@med.usc.edu (A.P.); ethan.layne@med.usc.edu (E.L.); cganjavi@usc.edu (C.G.); adityamd@usc.edu (A.D.); gmiranda@med.usc.edu (G.M.); jie.cai@med.usc.edu (J.C.); vasileios.magoulianitis@med.usc.edu (V.M.); karan.gill@med.usc.edu (K.G.); gerhard.fuchs@med.usc.edu (G.F.); mihir.desai@med.usc.edu (M.D.); igill@med.usc.edu (I.G.); 2 Artificial Intelligence Center at USC Urology, Los Angeles, CA 90033, USA

**Keywords:** readability, lay summary, clinical trials, patient education, Pub2Post, Generative Artificial Intelligence, artificial intelligence, LLM

## Abstract

Accessible health information is essential to promote patient engagement and informed participation in clinical research. Brief summaries on ClinicalTrials.gov are indented for lay people; however they are often written at a reading level that is too advanced for the public. This study evaluated the performance of a Generative Artificial Intelligence (GAI)-powered tool—Pub2Post—in producing readable and complete layperson brief summaries for urologic oncology clinical trials. Twenty actively recruiting clinical trials on prostate, bladder, kidney, and testis cancers were retrieved from ClinicalTrials.gov. For each, a GAI-generated summary was produced and compared with its publicly available counterpart. Readability indices, grade-level indicators, and text metrics were analyzed alongside content inclusion across eight structural domains. GAI-generated summaries demonstrated markedly improved readability (mean FRES 73.3 ± 3.5 vs. 17.0 ± 13.1; *p* < 0.0001), aligning with the recommended middle-school reading level, and achieved 100% inclusion of guideline-defined content elements. GAI summaries exhibited simpler syntax and reduced lexical complexity, supporting improved comprehension. These findings suggest that GAI tools such as Pub2Post can generate patient-facing summaries that are both accessible and comprehensive.

## Simple Summary

This study showed that an AI tool called Pub2Post can create short and clear health trial stories in plain words. It included short notes from health studies on cancers of the bladder, kidney, testis, and prostate. These stories were easier to read than the old ones on ClinicalTrials.gov and match the reading level of most middle school kids. They used simple words and reported all the key facts needed for someone to understand the study. This may help more people know about and join in in cancer research as well as make better notes for health studies in the future.

## 1. Introduction

Healthcare information is often too complex for the general public to read, leading to a major issue of understandability of medical content [1]. Recognizing the importance of delivering accessible health literacy, organizations such as the American Medical Association (AMA) and the European Union (EU) have issued guidelines aimed at improving the readability and accessibility of medical content [2,3].

In the wake of the COVID-19 pandemic, the public’s reliance on digital sources for health information surged, leading to digitalization of the population [4]. These sources include the internet and social media, which pose significant risks as they can rapidly spread misinformation [5]. To gather information from ongoing or recruiting clinical trials, physicians and the public alike often turn to platforms such as Clinicaltrials.gov which plays a key role by providing brief layperson summaries intended for delivering lay people information about the trial. A “brief summary” is intended as a short description of the clinical trial, including core descriptive information about a clinical trial—structured to summarize its purpose, design, population, and main questions, written in language intended for the lay public [6]. However, evidence suggests that these lay summaries are frequently falling short of the recommended readability marks for lay audiences [7].

Generative Artificial Intelligence (GAI) has shown to be a promising tool that can transform the way health information is delivered to the public [8,9,10,11,12,13]. GAI can be utilized to create these brief summaries intended for lay audiences that have an appropriate reading level without compromising necessary information [14]. The aim of this study is to evaluate the performance of a generative AI–powered tool in producing readable and comprehensive layperson-tailored summaries of clinical trials in urological oncology.

## 2. Materials and Methods

### 2.1. Clinical Trials Lay Summary Selection and Cohort

Urologic-oncology clinical trials were extracted from ClinicalTrials.gov using disease-specific search terms including “prostate cancer”, “bladder cancer”, “kidney cancer”, and “testis cancer”. Searches were performed independently for each disease. To identify currently active studies, the “recruiting” filter was applied to each search, isolating trials actively enrolling participants. The filtered results were then sorted from newest to oldest, and the top 50 most recent trials for each oncologic disease were extracted on 19 September 2025. From each disease-specific dataset, five trials were randomly selected using the RAND function in excel. Eligible trials were required to contain both a “Brief Summary” and “Detailed Description” section, and the respective oncologic disease term (prostate cancer, Bladder Cancer, Renal Cancer or Testis Cancer) had to be included in the trial’s “keywords” section. This process yielded 20 clinical trials (five per disease) for subsequent readability and content evaluation. This study is part of the Bridging Readable and Informative Dissemination with GenerativE Artificial Intelligence (BRIDGE-AI) initiative (https://osf.io/8yz6d/(accessed on 6 October 2025)).

### 2.2. GAI-Assisted Clinical Trial Lay Summary Generation

To generate the GAI-derived lay summaries, a newly implemented option within the Pub2People function of Pub2Post (www.pub2post.com (accessed on 29 September 2025) was utilized. Pub2Post is a GAI–powered tool that has been evaluated in prior research, demonstrating excellent performance in generating media content [15] and concise summaries [14] from existing medical literature. The system required no manual prompting and utilized dual-source trial data. For each of the 20 selected trials, the GAI model received two separate input files: one “Main View” file replicating the publicly accessible ClinicalTrials.gov trial page, and one “Researcher View” file containing expanded study details from backend trial documentation. Using these paired inputs, the GAI system autonomously produced a new brief summary for each clinical trial. This process was repeated five times per cancer type, resulting in 20 GAI-generated summaries across all disease cohorts.

### 2.3. Readability Scores, Grade Level Indicators and Text Metrics

Readability scores (RSs), grade-level indicators (GILs), and textual metrics (TMs) were calculated for both publicly available and GAI-generated brief summaries using a previously validated online tool (https://www.webfx.com/tools/read-able/ (accessed on 20 September 2025)). The validated readability measures (RSs and GILs) included the Flesch-Kincaid Reading Ease Score (FRES), Flesch-Kincaid Grade Level (FKGL), Gunning Fog Score (GFS), SMOG Index (SI), Coleman-Liau Index (CLI), and Automated Readability Index (ARI). Text metrics (TMs) such as total word count, sentence count, complex word count, percentage of complex words, average words per sentence, and average syllables per word were also collected.

### 2.4. Completeness Assessment of Brief Summaries

To assess content inclusion, all 40 brief summaries (20 publicly available and 20 GAI-generated) were compiled into a single Excel database. Using the “RAND()” function (Microsoft Excel, Redmond, WA, USA, 2024), summaries were randomly distributed between two independent raters (researchers at the University of Southern California with a B.S. and MD), each receiving 20 summaries representing a randomized mixture of publicly available and GAI-generated content. To reduce the possibility of any rater bias in the AI-generated brief summaries, the headings were removed and only the text was retained for the rater assessment. Raters were instructed to evaluate each summary against eight pre-specified content criteria corresponding to ClinicalTrials.gov structural sections. These included the Study Overview (Description), Study Overview (Conditions), Eligibility Criteria (Inclusion Criteria), Eligibility Criteria (Exclusion Criteria), Study Plan (Design Details), Study Plan (Arms and Interventions), Study Plan (Primary Outcome Measures), and Study Plan (Secondary Outcome Measures). Each domain was assessed dichotomously, with a score of 1 indicating presence and 0 indicating absence. Raters were also provided with direct links to each trial during the evaluation.

### 2.5. Statistical Analysis

Comparisons for all the readability indices (RSs, GILs, and TMs) between GAI-generated and publicly available brief summaries were treated as continuous variables and were compared with a Kruskal–Wallis Test. Fisher’s Exact Test was used for the categorical content inclusion metrics representing the presence or absence of specific study components. All analyses were performed using SAS (SAS Institute Inc., Cary, NC, USA, Version 9.4), with statistical significance defined as *p* < 0.05.

## 3. Results

### 3.1. Readability and Text Metric Comparison

**Figure 1:**
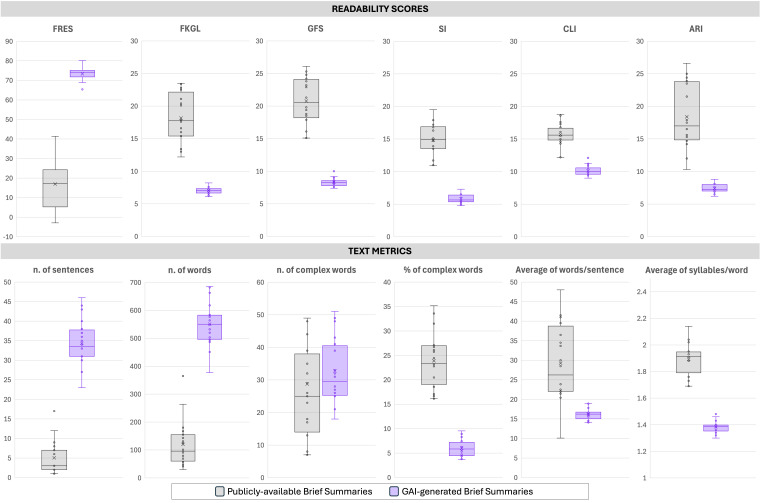
Comparative analysis of readability and text complexity metrics between Publicly available Brief Summaries and GAI-generated Brief Summaries from registered clinical trials. The upper panel reports six readability indices: Flesch Reading Ease Score (FRES), Flesch–Kincaid Grade Level (FKLG), Gunning Fog Score (GFS), Smog Index (SI), Coleman–Liau Index (CLI), and Automated Readability Index (ARI). The lower panel presents text-level measures, including the number of sentences, number of words, number and percentage of complex words, mean words per sentence, and mean syllables per word. GAI-generated Brief Summaries exhibited markedly higher readability scores and longer outputs, with significant differences indicated (*p* < 0.001 unless otherwise specified).

Readability indices and structural text metrics demonstrated statistically significant improvements in favor of GAI-generated brief summaries across all measures (Table 1 and Figure 1). GAI-generated summaries achieved a substantially higher FRES (mean 73.3 ± 3.5; median 74.2 [72.1–75.1]) compared with publicly available summaries (mean 17.0 ± 13.1; median 17.2 [5.4–23.9]; *p* < 0.0001), indicating markedly greater readability. Corresponding grade-level mean indices showed significant reductions for GAI-generated summaries: Flesch-Kincaid Grade Level (7.0 ± 0.5 vs. 18.2 ± 3.8), Gunning Fog Score (10.1 ± 0.8 vs. 15.7 ± 1.7), SMOG Index (8.3 ± 0.6 vs. 20.8 ± 3.5), Coleman-Liau Index (5.8 ± 0.7 vs. 14.8 ± 2.4), and Automated Readability Index (7.4 ± 0.7 vs. 18.4 ± 5.0), all *p* < 0.0001. These results indicate that GAI-generated texts were consistently written at a middle school reading level—as per AMA and EU requirements—whereas publicly available summaries required a much more complex reading ability. GAI-generated summaries were substantially longer, averaging 34.4 ± 5.8 sentences and 551.1 ± 76.2 words, compared with 5.1 ± 4.3 sentences and 120.3 ± 81.9 words in publicly available summaries (both *p* < 0.0001). Despite increased length, GAI outputs exhibited simpler structure, with shorter average sentence lengths (16.2 ± 1.4 vs. 29.3 ± 9.8 words per sentence, *p* < 0.0001) and lower syllabic density (1.4 ± 0.0 vs. 1.9 ± 0.1 syllables per word, *p* < 0.0001). Absolute counts of complex words were slightly higher in GAI-generated summaries (32.8 ± 9.7) compared with public summaries (28.8 ± 21.0; *p* = 0.0903, not significant). However, the percentage of complex words was significantly lower in GAI outputs (6.0% ± 1.8%) relative to publicly available summaries (23.9% ± 5.5%; *p* < 0.0001), reflecting simpler lexical composition.

### 3.2. Content Coverage of Publicly Available vs. GAI-Generated Brief Summaries

**Figure 2:**
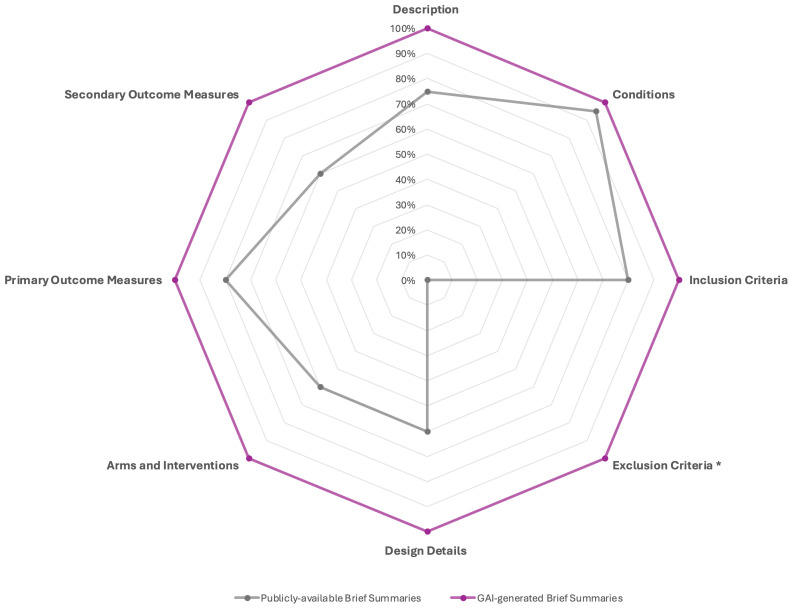
Comparative content inclusion analysis between Publicly available Brief Summaries and GAI-generated Brief Summaries from registered clinical trials. The radar chart illustrates the proportion of summaries containing key reporting elements across eight content domains: Description, Conditions, Inclusion Criteria, Exclusion Criteria, Design Details, Arms and Interventions, Primary Outcome Measures, and Secondary Outcome Measures. GAI-generated Brief Summaries consistently demonstrate more comprehensive coverage across all domains, reaching complete inclusion in all categories, whereas publicly available summaries show variable completeness, particularly under-reporting Design Details, Arms and Interventions, and Exclusion Criteria. * The exclusion criteria were reported by 19/20 trials included in the study.

Comparison of content elements between publicly available brief summaries and GAI-generated brief summaries revealed significant differences across multiple study components (Table 2 and Figure 2). While both formats consistently included conditions (95% vs. 100%, *p* = 1.00), GAI-generated summaries achieved complete coverage (100%) for all other sections, whereas public summaries showed variable inclusion rates. Within the Study Overview, description fields were present in 75% of public summaries compared with 100% of GAI-generated summaries (*p* = 0.05). Under Eligibility Criteria, inclusion criteria were reported in 80% of public summaries and 100% of GAI summaries (*p* = 0.11), whereas exclusion criteria were completely absent in the public summaries (0%) but present in all GAI-generated ones (100%), representing a highly significant difference (*p* < 0.0001). Within the Study Plan category, all design elements—design details, arms and interventions, and secondary outcome measures—were reported in 100% of GAI-generated summaries but only in 60% of public summaries (all *p* = 0.0033). Similarly, primary outcome measures were noted in 80% of public summaries and 100% of GAI summaries (*p* = 0.11).

## 4. Discussion

The present study evaluated the readability and content inclusion of publicly available brief summaries from clinical trials in urologic oncology compared with those generated by a GAI-powered tool—Pub2Post (www.pub2post.com). Using validated readability indices, GAI-generated summaries demonstrated significantly superior readability (all *p*-values < 0.001) while achieving complete inclusion of the elements outlined in the ClinicalTrials.gov guidelines. These findings highlight the potential of GAI to generate brief summaries that are both more accessible and more comprehensive, thereby improving public understanding and trust in clinical trial information. The internet has become an immense reservoir of medical information. In the wake of widespread digitalization—accelerated by the COVID-19 pandemic—it has become imperative to ensure that the health information consumed by the public is both verifiable and readable [4]. Health-related searches account for a substantial portion of online activity. However, prior studies have shown that such content is often either verifiable but not readable or readable but not verifiable [16]. Within this context, misinformation—especially via “Dr. Google” and social media—remains pervasive and can serve as a patient’s first encounter with their disease, including urologic malignancies. This underscores the importance of directing patients and the public to validated, trustworthy sources such as ClinicalTrials.gov [17,18]. When individuals visit ClinicalTrials.gov, the brief summary is the first section encountered. According to the ClinicalTrials.gov “Plain Language Guide,” this section should be written in simple, accessible language that allows the general public to easily understand a study’s goals, research questions, and design [6]. Our analysis revealed that the original brief summaries showed a collegiate reading level (18–24 years), far exceeding the recommended 6th–8th grade level. Such complexity poses a substantial barrier for patients seeking to comprehend clinical trial information, potentially resulting in confusion, anxiety, or reluctance to participate in research. Furthermore, lower readability may inadvertently increase physician workload, as patients turn to clinicians for clarification. Even for healthcare providers, overly complex text can hinder efficient communication, limiting their ability to translate trial information into patient-centered discussions. Beyond readability, our findings indicate that publicly available summaries frequently fail to meet content inclusion guidelines [7]. Considerable heterogeneity was observed in length—ranging from a single sentence to seventeen—yet many omitted key elements such as eligibility criteria, study design details, interventions, and outcome measures. Notably, none included exclusion criteria. These omissions compromise the completeness and transparency of trial information, reducing the utility of ClinicalTrials.gov as a comprehensive patient-facing resource.

By contrast, GAI-generated summaries addressed both readability and completeness shortcomings. The model consistently produced outputs at the recommended middle-school reading level (FRES 73.3; FKGL 7.0; GFS 10.1; SMOG 8.3; CLI 5.8; ARI 7.4) while fully incorporating all guideline-specified content elements. Although GAI summaries were longer (mean 34.4 sentences; 551 words), they exhibited simpler sentence structure, reduced lexical complexity, and lower syllabic density—features conducive to improved comprehension. Collectively, these characteristics underscore GAI’s ability to synthesize clinical information into clear, accurate, and lay-friendly summaries [14].

The GAI tool used for the purposes of this study (Pub2Post) has been validated multiple times to ensure that the information extracted from scholarly articles is accurate [14,15,19,20]. For example, a recent publication assessed the performance of Pub2Post in generating summaries tailored for patients from urology literature, finding accuracy, completeness, and clarity of information to range between 85 and 100% [14]. This highlights how a generative AI tool specifically tailored for this purpose can achieve higher performance compared to random prompting, which, because of the stochastic nature of GAI models, can be significantly lower [21]. Of note, Pub2Post also features a built-in fact-checking system that finds inaccuracies against the source, with a detection accuracy of 98.2% [20]. However, even if the performance of this GAI tool is impressive and consistent, human oversight is mandatory at this stage not only for detecting possible hallucinations and discrepancies between original sources and generated texts but also for identifying information that is clinically meaningful.

The broader implication of these findings extends beyond urologic oncology. Integrating GAI-based tools such as Pub2Post into clinical trial registries could enhance health literacy, promote patient engagement, and foster trust in evidence-based medicine. By providing complete, readable, and verified trial information in a single platform, GAI may also help mitigate the spread of misinformation and support equitable access to research participation opportunities.

This study has several limitations. First, the sample size was modest (*n* = 40), comprising 20 publicly available and 20 GAI-generated summaries, which may limit generalizability. Future research should include larger and more diverse disease cohorts to validate these findings. Second, while readability was measured using a validated tool (WebFX), the accuracy, clarity, and contextual appropriateness of each section’s content were not assessed. Establishing standardized tools or expert review frameworks to assess these dimensions will be crucial in future evaluations. Finally, the study did not examine user-centered outcomes—such as patient comprehension, satisfaction, or trust—which require a further dedicated investigation.

## 5. Conclusions

The GAI-powered tool assessed in the present study significantly and meaningfully improved both the readability and completeness of clinical trial brief summaries. Implementing such tools within ClinicalTrials.gov and similar registries could represent a critical step toward improving public access to comprehensible and accurate health information, thereby improving the lay public’s accessibility to health literacy and supporting informed participation in clinical trials.

## Figures and Tables

**Table 1:** Comparison of Readability Scores and Text Metrics between Publicly available Brief Summaries and GAI-generated Brief Summaries from registered clinical trials.

	Publicly Available Brief Summaries		GAI-Generated Brief Summaries		*p*-Value
**Readability Scores**	**mean (SD)**	**Median (IQR)**	**mean (SD)**	**Median (IQR)**	
Flesch Kincaid Reading Ease	17.0 (13.1)	17.2 [5.4–23.9]	73.3 (3.5)	74.2 [72.1–75.1]	*p* < 0.0001
Flesch Kincaid Grade Level	18.2 (3.8)	17.8 [15.4–21.8]	7.0 (0.5)	7.1 [6.7–7.3]	*p* < 0.0001
Gunning Fog Score	15.7 (1.7)	15.6 [14.9–16.6]	10.1 (0.8)	10.0 [9.6–10.6]	*p* < 0.0001
Smog Index	20.8 (3.5)	20.6 [18.3–24.0]	8.3 (0.6)	8.3 [7.9–8.6]	*p* < 0.0001
Coleman Liau Index	14.8 (2.4)	14.9 [13.6–16.9]	5.8 (0.7)	5.7 [5.4–6.3]	*p* < 0.0001
Automated Readability Index	18.4 (5.0)	17.0 [15.0–23.7]	7.4 (0.7)	7.3 [7.0–7.9]	*p* < 0.0001
**Text Metrics**					
Sentences	5.1 (4.3)	3.0 [2.0–7.0]	34.4 (5.8)	33.5 [31.0–37.5]	*p* < 0.0001
Words	120.3 (81.9)	96.0 [63.0–155.0]	551.1 (76.2)	550.5 [498.5–581.5]	*p* < 0.0001
Complex Words	28.8 (21.0)	25.0 [15.0–37.0]	32.8 (9.7)	29.5 [25.5–40.0]	0.0903
% Of Complex Word	23.9 (5.5)	23.3 [19.5–26.9]	6.0 (1.8)	5.9 [4.6–7.0]	*p* < 0.0001
Average Words Per Sentence	29.3 (9.8)	26.3 [22.1–38.0]	16.2 (1.4)	16.2 [15.2–16.7]	*p* < 0.0001
Average Syllables Per Word	1.9 (0.1)	1.9 [1.8–1.9]	1.4 (0.0)	1.4 [1.4–1.4]	*p* < 0.0001

This table encompasses readability scores, grade-level indicators, and additional text-level metrics, including average sentence length, total word count, syllable count per word, and the proportion of complex words. Readability scores measure how easily a text can be understood, with higher values indicating greater accessibility. In contrast, grade-level indicators operate inversely, where lower values reflect higher accessibility. Supporting statistics such as sentence length and word complexity offer further insight into the linguistic characteristics of the texts. GAI-generated summaries demonstrate enhanced readability (*p* < 0.0001 across all indices, except number of complex words), reflecting higher readability scores, lower grade levels, reduced syntactic complexity, and higher accessibility.

**Table 2:** Comparison of Content Inclusion Parameters between Publicly available Brief Summaries and GAI-generated Brief Summaries from Registered Clinical Trials.

		Publicly Available Brief Summaries	GAI-Generated Brief Summaries	*p*-Value
**Study Overview**	Description, No. (%)	15 (75)	20 (100)	0.05
	Conditions, No. (%)	19 (95)	20 (100)	1.00
**Eligibility Criteria**	Inclusion Criteria, No. (%)	16 (80)	20 (100)	0.11
	Exclusion Criteria, No. (%)	0 (0)	19 (100) *	<0.0001
**Study Plan**	Design Details, No. (%)	12 (60)	20 (100)	0.0033
	Arms and Interventions, No. (%)	12 (60)	20 (100)	0.0033
	Primary Outcome Measures, No. (%)	16 (80)	20 (100)	0.11
	Secondary Outcome Measures, No. (%)	12 (60)	20 (100)	0.0033

This table presents the proportion of summaries containing key reporting elements across three major domains: Study Overview, Eligibility Criteria, and Study Plan. Each domain includes specific content parameters (e.g., Description, Conditions, Inclusion/Exclusion Criteria, Design Details, Arms and Interventions, and Outcome Measures). Percentages represent the proportion of summaries incorporating each element. Statistical comparisons (*p*-values) indicate significant differences in content inclusion rates, with GAI-generated summaries demonstrating markedly higher completeness across most parameters (*p* < 0.05), particularly for Exclusion Criteria, Design Details, Arms and Interventions, and Secondary Outcome Measures. * The exclusion criteria were reported by 19/20 trials included in the study.

## Data Availability

The data presented in this study are available in the article and its Supplementary Materials. All AI-generated summaries and associated datasets used for analysis are provided as part of the Supplementary Files.

## Supplementary Materials

The following supporting information can be downloaded at: https://www.mdpi.com/article/10.3390/curroncol32120696/s1, Table S1. Clinical Trials selected with the original brief summary and the corresponding generated GAI brief summary. Table S2. Definitions for Independent Assessment from ClinicalTrials.gov Submission Process.

## Abbreviations

The following abbreviations are used in this manuscript:

GAI	Generative Artificial Intelligence
AMA	American Medical Association
EU	European Union
BRIDGE-AI	Bridging Readable and Informative Dissemination with GenerativE Artificial Intelligence
RSs	Readability Scores
GILs	Grade Level Indicators
TMs	Text Metrics
