# Supplementary material for: Readability Optimization of Layperson Summaries in Urological Oncology Clinical Trials: Outcomes from the BRIDGE-AI 8 Study
Source: Curr Oncol. 2025 Dec 10;32(12):696. doi: 10.3390/curroncol32120696 (PMC12731858; doi:10.3390/curroncol32120696)
Supplement: Supplementary file 1 [file curroncol-32-00696-s001.zip › curroncol-3950567-supplementary.pdf]

Supplementary

Table S1. Clinical Trials selected with the original brief summary and the corresponding generated GAI brief summary.

Cancer Type	Count	NCT Number	Study Title	Study URL	Brief Summary	GAI generated summary
Prostate	1	NCT07182279	Neoadjuvant High Dose Rate Brachytherapy Prior to Radical Prostatectomy in Patients With Prostate Cancer	https://clinicaltrials.gov/study/NCT07182279	"This is a Phase I/II trial evaluating the effectiveness of adding neoadjuvant HDR-B prior to RALP for HR-PCa patients with selective AAB for decipher high risk or pathologically node positive patients.<br><br>Patients with newly diagnosed, histologically confirmed, non-metastatic, HR-PCa who are scheduled to receive RALP will be eligible to participate in the study."	Study Overview<br>This study looks at a new way to treat men with a type of prostate cancer called high-risk prostate cancer. The doctors want to test if giving radiation inside the body before surgery is safe and works well. This kind of radiation is called high dose rate brachytherapy. The men in the study will get this treatment first, then have surgery to remove the prostate. Some men, who have higher cancer risk based on lab tests or who have cancer in their lymph nodes, will get extra medicine after surgery. The main goal is to see if this plan is safe and doable. The study will follow each man for about two years after surgery.<br>Eligibility Criteria<br>Only men who are at least 40 years old and have prostate cancer proven by a tissue test can join. They must not have cancer that has spread far in the body. They need to be healthy enough to have both surgery and the special radiation. They also need to meet at least one high-risk sign like high PSA levels, a high Gleason score, a big tumor, or several cancer spots in the biopsy. They cannot join if they had major cancer in the last few years (except some skin cancers), if they have had other radiation to the pelvis, or if they used certain hormone treatments in the past six months. Also, if their prostate is too large or they have high bladder symptoms, they cannot join.<br>Study Plan Design Details<br>This study will not use random choice. All the men will get the same main treatment. The main goal is to treat the cancer. Everyone will know what treatment they are getting. There are two phases to test both safety and how well the treatment works. The study will take place at one hospital in Texas.

						Arms and Interventions<br>All men in the study will get one dose of high dose rate brachytherapy inside the prostate. This will happen about one to two months before surgery. After surgery, if the lab tests show high risk or if cancer is found in the lymph nodes, the man may also get hormone-blocking pills for about three months. These pills help stop the cancer from growing by blocking male hormones. Some men may also get radiation to the pelvis after surgery if cancer is found in the lymph nodes. Which hormone pills are given will depend on the doctor's choice. The type of hormone pill could vary from one patient to another.<br>Primary Outcome Measures<br>The study will first check if this plan of treatment is safe. Doctors will look to see if there are bad side effects. They will also check if the men finish all the treatments they are supposed to get. These checks will happen over the two years after surgery.<br>Secondary Outcome Measures<br>The study will also look at how this treatment affects the men's daily life and symptoms. Men will fill out forms about how they feel and how their bodies are working at different times over two years. Doctors will also see how the cancer responds by checking blood tests, MRI scans, and the prostate after surgery. They will check if the cancer comes back by testing a blood marker called PSA. If the PSA goes up again, it may mean the cancer is back. They will also check if the cancer spreads to other parts of the body by using scans or other tests if needed.
Prostate	2	NCT07179783	Sacituzumab Tirumotecan in Combination With Tagitanlimab in the Treatment of Aggressive Variant Prostate Cancer (AVPC) and Neuroendocrine Prostate Cancer (NEPC)	https://clinicaltrials.gov/study/NCT07179783	This study is a prospective, single arm II clinical trial. The main objective of the study is to evaluate the efficacy and safety of the combination of Sacituzumab Tirumotecan (SKB264) and Tagitanlimab (KL-A167) in the treatment of AVPC (aggressive variant prostate cancer) and NEPC (neuroendocrine prostate cancer).	Study Overview (Detailed Description and conditions)<br>This study tests a new drug mix for two rare types of prostate cancer that grow and spread fast. These types are called aggressive variant prostate cancer and neuroendocrine prostate cancer. These cancers are hard to treat and most people do not live long after they are found. There are no standard drugs that work well for them. Right now, doctors use drugs like those used for a kind of lung cancer

called small cell lung cancer. This study will try a mix of two drugs, called Sacituzumab Tirumotecan and Tagitanlimab. Both aim to slow the cancer and help the immune system fight it. The study will test how safe this mix is and if it helps people live longer.

Eligibility Criteria (Inclusion Criteria and Exclusion Criteria)

To join, a person must be a man age 18 or older. He must have one of the two rare types of prostate cancer proven by lab tests. He must have tried some other drugs before and the cancer must have gotten worse. He must still have a good level of strength and at least one tumor that can be measured. His body's blood, liver, and kidney tests must be in safe ranges. He must not have any other serious health issues like bad heart problems, lung disease, or brain tumors. He cannot have had some types of cancer drugs before, like drugs that change how the immune system works. He must not have had a major surgery or serious infection lately. He must not have diseases like HIV or hepatitis, and cannot be allergic to the drugs in the study.

Study Plan Design Details

This is a phase two study, which means it tests how well the drug mix works and if it is safe. Everyone in the study gets the same treatment. There is no sugar pill or other group to compare. No one is kept in the dark about the treatment they get. The goal is to see if this mix can help people with these hard-to-treat prostate cancers.

Arms and Interventions

All patients in the study will get both Sacituzumab Tirumotecan and Tagitanlimab. These are given through a drip into a vein every two weeks. People will keep getting the drugs until their cancer grows again, they feel too sick from side effects, or they choose to stop. The most someone can get Tagitanlimab is for two years. If needed, doctors may lower the drug dose to help manage side effects.

Primary Outcome Measures

The main thing the study looks at is how many

						people have their tumors shrink or disappear after getting the drug mix. This is called the response rate. Doctors will use scans and tests to check if the treatment is working over a span of up to two years.<br>Secondary Outcome Measures<br>Other things doctors will check include how long it takes before the cancer grows again, how long the good response lasts, and how long each person lives from the start of treatment. They will also see how many people feel bad effects from the drugs and how bad those effects are. Doctors will also look at signs in the body, like certain proteins, that may help tell who will do better with the treatment.
Prostate	3	NCT07181473	A Study to Evaluate the Safety, Pharmacokinetics and Efficacy of TJ101 in Patients With Advanced/Metastatic Solid Tumors	https://clinicaltrials.gov/study/NCT07181473	"The goal of this clinical trial is to evaluate whether TJ101, an investigational antibody-drug conjugate (ADC), can safely and effectively treat patients with advanced solid tumors.<br><br>The main objectives of this study are :<br><br>* To Determine the maximum tolerated dose (MTD) and recommended dose for expansion (RDE) of TJ101<br>* to show preliminary antitumor activity in patients with advanced solid tumors<br><br>Participants will:<br><br>* Receive intravenous (IV) infusions of TJ101 at escalating dose levels (during dose escalation) or at the selected expansion dose.<br>* Undergo regular tumor imaging to assess response.<br>* Provide blood samples for pharmacokinetics (PK) and biomarker analysis.<br>* Be monitored for side effects and overall tolerability.<br><br>This study is being conducted in adult patients with advanced or metastatic solid tumors who have exhausted standard treatment options"	Study Overview (Detailed Description and Conditions):<br>This study is testing a new drug called TJ101 to see if it is safe and helps people with cancer. TJ101 is a type of antibody-drug conjugate, which is a special drug that finds and attacks cancer cells. The study includes adults who have serious cancers that have spread and cannot be cured with usual treatments. The drug will be given through a vein every few weeks. The study will check if the drug is safe, how it moves in the body, and if it helps shrink tumors. Some people will get different doses to find the best one. The study looks at cancers like lung, prostate, and food pipe cancer.<br>Eligibility Criteria (Inclusion Criteria and Exclusion Criteria):<br>People can join if they are adults and have a type of solid cancer that has grown or spread. They must have tried other treatments that did not work or have no other options. Their health should be good enough to join, including having working organs and enough blood cells. People cannot join if they had some types of cancer drugs before, have certain heart or lung problems, or are very sick with infections. Pregnant or breastfeeding women also cannot join. People must agree to use birth control if they can have children.<br>Study Plan Design Details:<br>This is a phase one study, which means it is the first time the drug is being tested in people. Everyone

						will know what they are getting. In the first part, groups will get small doses to see how much the body can take. If the drug looks safe, more people will get a steady dose to see how well it works. The drug is given once every three weeks. People will keep getting it until their cancer gets worse, the side effects are too bad, or they choose to stop.<br>Arms and Interventions:<br>There is only one group in this study. Everyone in the group will get TJ101 through a vein. At first, doses will go up in small steps to see what amount is safe. Later, more people will get the chosen best dose. TJ101 is made to target certain parts of cancer cells to kill them while hurting less of the rest of the body.<br>Primary Outcome Measures:<br>The study will first look at how many people have bad side effects after getting the drug. These side effects may include low blood cells, fever, bleeding, or other serious problems. It will also count how many people get sick from the drug, how long the side effects last, and if any are caused by the treatment.<br>Secondary Outcome Measures:<br>The study will also check how well the drug works. It will look at how many people have their cancer shrink or stay the same, how long that lasts, and how long people live without the cancer getting worse. The study will also look at how much drug is in the blood and how long it stays in the body. It will check if the body makes antibodies against the drug, which might stop it from working.
Prostate	4	NCT07172685	Study on Triple Therapy Combined With HIFU for High-Tumor-Burden mHSPC	https://clinicaltrials.gov/study/NCT07172685	"This study is a single-arm prospective cohort study designed to evaluate the efficacy and safety of triple therapy (ADT + darolutamide + docetaxel) combined with transrectal high-intensity focused ultrasound (HIFU) focal therapy in patients with high-tumor-burden metastatic hormone-sensitive prostate cancer (mHSPC).<br><br>A total of 116 high-tumor-burden mHSPC patients will be enrolled and are scheduled to receive the following treatment:	Study Overview (Detailed Description and Conditions)<br>This study looks at men who have a type of prostate cancer that has spread and still responds to hormone treatment. This is called hormone-sensitive prostate cancer. These men have a high number of cancer spots in the body. The goal is to test if giving three types of medicine along with a sound-wave treatment can help more than using medicine alone. The three types of medicine are:<br>ADT, or androgen deprivation therapy, which

	Darolutamide + Docetaxel + ADT + Transrectal HIFU Focal Therapy for the Prostate."	lowers male hormones.<br>Darolutamide, which blocks the male hormone signal in cancer cells.<br>Docetaxel, which is a type of cancer-killing drug.<br>The extra treatment is called HIFU, which stands for high-intensity focused ultrasound. This is a type of sound wave that heats and destroys cancer in the prostate. The HIFU is done through the rectum, which is the last part of the large bowel.<br>This study will follow about one out of one hundred men who have this kind of cancer. It will watch how they do after this treatment. The hope is to slow the spread of cancer, help men live longer, and reduce side effects from medicine .<br>Eligibility Criteria (Inclusion Criteria and Exclusion Criteria)<br>Men can join this study if they are over the age of eighteen and have prostate cancer that has spread to the bones or other body parts. Their cancer must still respond to hormone-lowering treatment. They must be in good health to handle cancer drugs. They must also agree to use birth control during the study.<br>Men cannot join if the cancer is in a spot that is hard to reach with the HIFU tool. Other reasons for not joining include having had some types of bowel surgery, having some types of heart or brain problems, or using other cancer drugs before. People who have infections or certain other health problems may also not be able to join.<br>This is to keep the study safe and make sure results are not confused by other health problems .<br>Study Plan Design Details<br>The study will not compare different groups. All men in the study will get the same treatment. This means everyone will take the three medicines and have the sound-wave therapy.<br>Doctors will check how well the treatment works and whether it causes any harm. The study will take place in China, and it will last for a few years. All care is open label, which means doctors and patients know what treatment is given .<br>Arms and Interventions

						All patients in the study will get the same care plan. They will take darolutamide pills two times each day. They will also get an injection of a hormone-lowering drug every three months. Every three weeks, they will get a drug called docetaxel through a drip. This will happen six times. They may also take other drugs to help with side effects.<br>In addition to the drugs, they will have HIFU, which uses strong sound waves to heat and destroy cancer in the prostate. This will be planned using body scans and a sample of the tumor. They may get this HIFU one or two times .<br>Primary Outcome Measures<br>The main thing the study looks at is how long the cancer stays under control after treatment. This is checked using body scans. The study will measure this over three years. They will also check how quickly prostate-specific antigen (PSA), a blood marker linked to prostate cancer, rises again after treatment.<br>Secondary Outcome Measures<br>The study also checks other things, like how many men have a strong drop in their PSA after treatment. PSA is a sign of how active the cancer is. If PSA drops a lot, it may mean the treatment worked well. They will look at this at three points in time: after three months, after six months, and after a year. They will also see how long it takes for the cancer to get worse or for the patient to pass away. The study will follow each person for up to two years after they start the medicine.
Prostate	5	NCT07168616	Study of PSMA PET/CT Imaging to Help Select Men With Low-Risk Prostate Cancer for Active Surveillance	https://clinicaltrials.gov/study/NCT07168616	This is a multicenter, prospective diagnostic accuracy study evaluating the Istanbul PSMA PET/CT Criteria (IPPC) for selecting patients with biopsy-confirmed low-risk prostate cancer (ISUP Grade 1) for active surveillance (AS). The study integrates delayed Ga-68 PSMA PET/CT imaging into the diagnostic pathway to refine patient stratification, minimize overtreatment and potentially reduce unnecessary biopsies and MRI, or exposing high-risk individuals to the danger of cancer progression if left untreated scans.	Study Overview (Detailed Description and Conditions)<br>This study looks at a new scan called PSMA PET/CT to help pick men with low-risk prostate cancer who might not need treatment right away. Prostate cancer grows in the male sex gland and is common. Some men have a slow type that may not need treatment. Others have a type that can spread fast. Today, doctors use tests like blood work, physical checks, and tissue samples to tell which kind a man has. But these tests do not always get it right. This study will use a new kind of scan to get a better look

at the cancer inside the prostate. This scan may help find the slow types and avoid extra tests. The scan will look at how much of a special dye the cancer absorbs. If the cancer absorbs very little, the man might be safe to watch without treatment. If it absorbs a lot, he may need more care. The study will check if this scan is better than older tests.

Eligibility Criteria (Inclusion Criteria and Exclusion Criteria)

Men who may join this study must have had a prostate scan in the last few months and a tissue sample that shows they have the slow type of prostate cancer. The cancer should be small, and the man should be in good health with many years ahead of him. Men cannot join if they have a fast-growing type of cancer, if they had prostate surgery, or if they had radiation near the prostate. Men with bad kidney health or who cannot follow up with doctor visits also cannot join. Only men who live in Turkey can take part in this study.

Study Plan Design Details

This study watches a group of men over time. It does not give them treatment. Instead, it follows them for two years. Doctors will check how well the new scan works to find the type of cancer that may need care. The study will not take blood or tissue for other research.

Arms and Interventions

All men in the study will get a delayed scan called Ga-68 PSMA PET/CT. This scan uses a small amount of dye that sticks to the cancer. Doctors wait two hours after the dye is given, then do the scan. They will check how bright the cancer shows up to decide if the man should be watched or treated.

Primary Outcome Measures

The study will see how well the scan finds the more dangerous kind of prostate cancer. Doctors will check how often the scan is right or wrong by comparing it to surgery results or health changes over two years. They will look at how good the scan is at telling which men have the more serious cancer and which do not.

Secondary Outcome Measures

						The study will also look at how many men can avoid getting another tissue test because of the scan. If the scan shows the cancer is likely slow, the man might skip extra tests. Doctors will also use the scan to help track the cancer during check-ups. They may do another scan each year or sooner if the cancer seems to change.
Bladder	1	NCT07176962	A Cell-free DNA Methylation Blood-Based Test for Biliary Tract Cancers Screening	https://clinicaltrials.gov/study/NCT07176962	Biliary tract carcinoma (BTC), including gallbladder cancer, intrahepatic cholangiocarcinoma, and extrahepatic cholangiocarcinoma, ranks sixth in incidence among gastrointestinal malignancies and tenth in cancer-related mortality worldwide. Due to the lack of specific early symptoms, high malignancy, and frequent recurrence and metastasis, the rate of curative resection is only about 16.5%, and the overall 5-year survival rate is less than 5%. Early and accurate detection is therefore critical for improving patient outcomes. Circulating tumor DNA (ctDNA), a fraction of circulating free DNA (cfDNA), carries genetic and epigenetic information from tumor cells and can be detected even at the early stages of cancer development. Among various liquid biopsy biomarkers, ctDNA methylation shows particular advantages in sensitivity and specificity for early cancer detection and monitoring. This study aims to evaluate the application of cfDNA methylation liquid biopsy in the diagnosis and management of BTC.	Study Overview (Detailed Description and Conditions)<br>This study looks at a kind of cancer that can grow in parts of the bile tract. This includes the gallbladder and tubes in and around the liver. These cancers grow fast and often come back after treatment. Most people do not have signs early on, so it is hard to find and treat the cancer in time. As a result, only a small number of people are able to get surgery to remove it, and very few survive five years. This study wants to test a new way to find this cancer early by looking for tiny bits of DNA in the blood that come from tumors. This DNA, called circulating tumor DNA or ctDNA, has special changes, like a process called methylation. Methylation is a change to the DNA that does not affect the code but still changes how it works. These changes can be spotted even before the cancer grows large. This test is a type of "liquid biopsy," which means it uses blood instead of surgery. It may help tell the difference between cancer and non-cancer problems in the bile tract and guide doctors in choosing treatment.<br>Eligibility Criteria (Inclusion Criteria and Exclusion Criteria)<br>The study is for people between young adults and older adults. People can join if they are healthy or have cancer or other belly-related illnesses. To join, people need to agree to take part and give blood. People with cancer must have proof of their cancer, like a lab report or scan. Their health must be good enough to safely join the study. People cannot join if they are pregnant, breastfeeding, have had a recent blood transfusion, have a history of cancer treatment, or have certain blood or immune problems. Healthy people must also not have serious health issues or past cancer. Each group

must follow strict rules, so the test results are clear and fair.

Study Plan Design Details

This is an "observational" study. That means the doctors will not give people new drugs or treatments. Instead, they will just collect and study blood samples. The goal is to compare groups. One group will have people with different types of bile tract cancer. Another group will have people who do not have this cancer but may have other belly or liver problems. There will also be healthy people with no illness. The blood samples will help doctors study the special tumor DNA and how it changes in cancer.

Arms and Interventions

The people in the study will be put into different groups. One group will include people with bile tract cancer, like gallbladder cancer or types that affect inside or outside the liver. Another group will include people with other stomach cancers. Some people will have non-cancer bile problems, like gallstones or infections. A separate group will be healthy people with no disease. No one will get new drugs. They will only give blood, and the researchers will study the DNA inside it.

Primary Outcome Measures

The main thing the study wants to check is how good this blood test is at finding cancer in the bile tract. The doctors will look at how often the test is right when it says someone has cancer (this is called sensitivity) and how often the test is right when it says someone does not have cancer (this is called specificity). They also want to know if the test is accurate overall.

Secondary Outcome Measures

Doctors also want to check if the test works just as well for different types of bile tract cancer, like those in the gallbladder or liver area. They will see if it works for early-stage and late-stage cancer. Another goal is to see if the test can tell the difference between cancer and other non-cancer bile problems. They will also watch how the test works over time. They want to know if the test can show if the cancer

						comes back after treatment or how long a person stays healthy. They will check this over many years after the first test.
Bladder	2	NCT07165236	Association of POCD With Circulating Biomarkers in Patients Undergoing TUR of Bladder Tumor	https://clinicaltrials.gov/study/NCT07165236	Bladder tumor is one of the most widespread tumors in the world, with increasing prevalence at the global level. One of the procedures in patients with bladder tumors is transurethral resection of the bladder (TURM) most often performed endoscopically under general anesthesia. This patient population has certain characteristics in common. One of them is exposure to common risk factors for the formation of bladder tumors, such as aniline dyes and solvents. These substances are associated with the onset of neurodegeneration and oxidative stress. Smoking is another factor that affects the formation of bladder tumor. A significant part of patients with bladder tumor are an elderly population, which is repeatedly exposed to surgical procedures, with numerous comorbidities, with a high risk of postoperative complications and the development of perioperative cognitive deficits, which can further complicate the postoperative course and further treatment. In the group of patients with a bladder tumor who will undergo TURM, no evaluation of risk factors related to perioperative cognitive deficit was performed, nor was there an examination of the perioperative cognitive deficit itself. In them, the identification of factors for postoperative cognitive disorder is essential. The concept of clinical frailty is becoming more important and relevant when providing healthcare services to patients. The use of the clinical frailty scale as a tool in clinical practice provides information on the adequate direction of care for patients. Decrease in muscle strength can lead to limitations in the functioning of a certain individuals. In recent years muscle strength has come to be a very important component of health, regardless of a person's age and clinical condition.The hand grip test is a test used to measure the maximum isometric strength of the hand and forearm muscles. The MMSE test and MoCA are the most frequently used methods in the	Study Overview (Detailed Description and Conditions)<br>This study looks at people who have a type of bladder cancer. They will have a surgery called transurethral resection of the bladder tumor. This is done with a camera and tools that go through the urine tube. Many people who get this surgery are older and may have other health problems. These people may have changes in thinking or memory after surgery. This is called perioperative cognitive disorder, or POCD. The study wants to learn what causes POCD. It will look at things in the blood that show brain changes or swelling, like S100B and neuron-specific enolase. It will also check how strong the patient's hand grip is. This test helps show how strong a person is overall. Two thinking tests called MMSE and MoCA will be used before and after surgery to check for changes in memory and thinking. The study will also look at body functions like liver and kidney health, and other blood tests. No one has looked at all these things together in people getting this surgery, so this study wants to fill that gap.<br>Eligibility Criteria (Inclusion Criteria and Exclusion Criteria)<br>People in this study must be between the ages of about twenty and eighty. They must have bladder cancer and be getting this planned surgery. They must be able to talk, read, and sign papers. People will not be in the study if they are younger than about twenty or older than eighty. Also, people cannot be in the study if they cannot talk or read, are in an emergency, or are allergic to study drugs. People in very bad health or with brain problems from birth will not be in the study either.<br>Study Plan Design Details<br>This is an observational study, which means the doctors will watch and collect data but will not change how patients are treated. It will look forward in time to see how patients do after surgery. The

					detection of cognitive impairment in clinical and research fields. In addition to laboratory indicators of organic function, circulating indicators of neuroinflammation, like S100B and neuron-specific enolase, will be correlated with the patient's cognitive status.So far, no research has been conducted on the dynamics of indicators of organic function, circulating indicators of neuroinflammation, perioperative cognitive changes and clinical frailty in patients undergoing bladder tumor operation.	study will include people getting the bladder surgery as planned. Doctors will follow them closely before and after surgery to see if thinking problems develop.<br>Arms and Interventions<br>There is just one group in this study. It includes all adults over twenty who are having this bladder surgery. The study does not give special treatment. It just watches and tests people. It will not include people who are too young, too old, very sick, or unable to understand the study.<br>Primary Outcome Measures<br>The main thing the study will check is if the patients' memory or thinking changes after surgery. This will be done using two tests. One is called the Mini-Mental State Exam, or MMSE. The other is called the Montreal Cognitive Assessment, or MoCA. These tests will be given before surgery, then again one day and two days after surgery. The scores will show if the patient’s thinking has stayed the same, gotten worse, or improved.<br>Secondary Outcome Measures<br>The study will also look at changes in some blood signs that may show brain changes. These include S100B and neuron-specific enolase, or NSE. It will also check other signs in the blood like IL4 and IL6. These are linked to swelling in the body. These blood tests will be done before surgery, two hours after, and one day after. This may help doctors learn what causes memory or thinking problems after surgery.
Bladder	3	NCT07118176	Determining the Biodistribution of an Imaging Tracer (68Ga-FAPi-46) in Patients With Solid Tumors or Hematologic Cancers	https://clinicaltrials.gov/study/NCT07118176	This phase I trial is evaluating a new imaging tracer (68Ga-FAPi-46) with positron emission tomography (PET)/computed tomography (CT) to determine where and to which degree the tracer (68Ga-FAPi-46) accumulates in normal and cancer tissues (the biodistribution) in patients with solid tumors or hematologic (blood) cancers. PET is an established imaging technique that utilizes small amounts of radioactivity attached to very minimal amounts of tracer, in the case of this research, 68Ga-FAPi-46. Because some cancers take up 68Ga-FAPi-46, it can be seen with PET. CT utilizes x-rays that traverse the	Study Overview (Detailed Description and Conditions)<br>This study is checking how a new scan tool called gallium Ga 68 FAPi-46 works in people who have different types of cancer. This tool helps doctors see where cancer is in the body. It uses a small amount of safe radiation. The scan is called PET/CT, which means it combines two kinds of pictures: one shows how the body works, and the other shows body parts clearly. The scan can help tell where the tracer goes in both healthy and cancer parts of the body. The types of cancer being studied include cancers of

body from the outside. CT images provide an exact outline of organs and potential inflammatory tissue where it occurs in a patient's body. Combining a PET scan with a CT scan can help make the image easier to interpret. PET/CT scans are hybrid scanners that combine both modalities into a single scan during the same examination.

the brain, breast, lung, skin, bone, stomach, liver, and more. It also includes blood cancers and cancers in glands like the prostate or thyroid.

Eligibility Criteria (Inclusion and Exclusion Criteria)

People can join if they have or are thought to have any of many kinds of cancer, like brain, lung, breast, skin, or blood cancers. They must be grown-ups and able to stay still during the scan. They must also agree to be in the study by signing a paper. People cannot join if they are pregnant or breastfeeding. They also cannot join if their health might make it hard to take good scan pictures.

Study Plan Design Details

This study is meant to help with finding cancer, not for treatment. Everyone in the study gets the same care. There is no hiding of who gets what — all patients and doctors know. Each person will get one scan with the new tracer. They may also get another scan with a tool already used in hospitals. This study is in early testing (called phase one) and will take place at a hospital in Los Angeles. About thirty people will join.

Arms and Interventions

All people in the study will get the tracer gallium Ga 68 FAPi-46 through a small tube in the arm. After that, they will get a scan called a PET/CT. This will happen about a half hour to an hour later and will take around half an hour. Some people may also get another scan using a different tracer called fludeoxyglucose F-18. Both tracers help take pictures of how cancer shows up in the body.

Primary Outcome Measures

The main goal is to see where the tracer gallium Ga 68 FAPi-46 goes in the body. This includes both normal and cancer parts. The scan will show how much of the tracer is taken in by these parts. Doctors will look at this over a two-year period.

Secondary Outcome Measures

Another goal is to compare this new tracer with a tracer already in use, called fludeoxyglucose F-18. This helps doctors see if the new tool works the same, better, or in different ways than the old one. They will look at patterns of how both tracers

						behave in the body to understand different types of cancer better.
Bladder	4	NCT07087860	Therapeutic Plasma Exchange With Enfortumab Vedotin and Pembrolizumab for Treatment of Bladder Cancers	https://clinicaltrials.gov/study/NCT07087860	This phase II trial compares therapeutic plasma exchange followed by enfortumab vedotin and pembrolizumab to standard of care next-line therapy for the treatment of patients with bladder or upper urinary tract cancers that have spread from where they first started (primary site) to other places in the body (metastatic) and that have not responded to previous treatment (refractory). TPE is a process that slowly removes a patient's blood through an intravenous or central line. The blood is sent through a machine that separates the plasma (the liquid part of blood) from other blood components (red cells, white cells, platelets). The plasma is then removed. The remaining blood components are combined with replacement fluid and returned to the patient's bloodstream through the intravenous or central line. Enfortumab vedotin is a monoclonal antibody, enfortumab, linked to an anticancer drug called vedotin. It works by helping the immune system to slow or stop the growth of cancer cells. Enfortumab attaches to a protein called nectin-4 on cancer cells in a targeted way and delivers vedotin to kill them. It is a type of antibody-drug conjugate. Immunotherapy with monoclonal antibodies, such as pembrolizumab, may help the body's immune system attack the cancer, and may interfere with the ability of tumor cells to grow and spread. Treatment with enfortumab vedotin and pembrolizumab is already approved by the Food and Drug Administration for the treatment of bladder cancer, but TPE is not. Combining TPE with enfortumab vedotin and pembrolizumab may work better than standard of care options for treating metastatic and refractory bladder and urinary tract cancers.	Study Overview (Detailed Description and Conditions)<br>This study is for people with bladder or upper urinary tract cancer that has spread and did not get better with past treatment. Doctors are testing a new way to help the body fight this type of cancer. The treatment uses a process called plasma exchange. This means some of your blood is taken out, the liquid part (called plasma) is removed, and the rest is put back in with new fluid. This is done before giving two drugs: enfortumab vedotin and pembrolizumab. These drugs help the body’s immune system find and kill cancer cells. The goal is to see if this new mix works better than the usual care. Usual care means the treatment a doctor would normally choose next. This study will help find out if the new way helps more people, helps them live longer, or causes fewer side effects.<br>Eligibility Criteria (Inclusion and Exclusion Criteria)<br>To join, you must be a grown-up. You must have bladder or upper tract cancer that came back or kept growing after treatment with enfortumab vedotin and pembrolizumab. Your cancer must be seen on scans. You need to feel well enough to take part. Your blood tests must look okay, and you must not be pregnant. You must say yes in writing, be ready to give blood and other samples, and be willing to go back to the clinic during the study. You cannot join if you are pregnant or nursing, or if you cannot use birth control. You also cannot join if you have a type of cancer cell not allowed in the study, or another kind of cancer that needs care. You may not join if you had heart problems in the past few months or have mental health or drug use issues that may stop you from taking part.<br>Study Plan Design Details<br>This is a treatment study. People will be picked by chance to get one of two treatments. The study is open, so both the patient and the doctor will know what treatment is being given. The goal is to see which works better. People will be in the study for

						several years, and the team will check on their health and how the cancer is doing.<br>Arms and Interventions<br>One group will get plasma exchange for a few days, followed by the drugs enfortumab vedotin and pembrolizumab through a vein. This happens in set cycles about every three weeks. They will also have blood and urine tests and scans like CT or MRI. The other group will get the treatment the doctor would usually choose. This group will also get tests and scans. Both groups may give samples to help learn more about how the treatment works.<br>Primary Outcome Measures<br>The main thing the study looks at is how many people the treatment helps. This means it checks if the cancer shrinks or goes away in people who take part. Doctors will look at this during the first few months after treatment.<br>Secondary Outcome Measures<br>The study also looks at how long the cancer stays gone before it grows again. It checks how long people live after the treatment. It keeps track of any side effects people have. It also asks how people feel during and after treatment using a set of questions about feeling weak or numb in hands or feet. This helps doctors learn about the impact of the treatment on daily life.
Bladder	5	NCT07061964	Combining Immunotherapy and Radiation Therapy to Help Patients Avoid Bladder Removal After Treatment Shrinks Muscle Invasive Bladder Cancer, BRIGHT Trial	https://clinicaltrials.gov/study/NCT07061964	This phase II trial tests the effect of giving pembrolizumab in combination with radiation therapy after chemotherapy in preventing surgery to remove the bladder in patients with muscle invasive bladder cancer. Standard of care therapy includes chemotherapy before surgery (neoadjuvant) to shrink or get rid of the tumor. Immunotherapy with monoclonal antibodies, such as pembrolizumab, may help the body's immune system attack the tumor, and may interfere with the ability of tumor cells to grow and spread. Radiation therapy uses high energy x-rays, particles, or radioactive seeds to kill cancer cells and shrink tumors. Photon beam radiation therapy is a type of radiation therapy that uses x-rays or gamma rays that come from a special machine called a linear	Study Overview (Detailed Description and Conditions):<br>This study looks at a new way to treat bladder cancer that has spread into the bladder muscle. It tries to help patients avoid having their bladder removed. People first get strong drugs, called chemotherapy, to shrink the cancer. Then, they get two other treatments: one is a kind of immune medicine called pembrolizumab, which helps the body fight cancer, and the other is radiation, which uses strong light rays to kill cancer cells. The hope is that this mix of treatments can stop the cancer without the need to remove the bladder. This study is for people with stage two or stage three bladder cancer that has not spread to other body parts.<br>Eligibility Criteria (Inclusion and Exclusion

	accelerator. The radiation dose is delivered at the surface of the body and goes into the tumor and through the body. Giving pembrolizumab in combination with radiation therapy after neoadjuvant chemotherapy may help prevent surgical removal of the bladder in patients with muscle invasive bladder cancer.	Criteria):<br>People can join this study if they are adults with a type of bladder cancer that grows into the muscle and has not spread. They must have had tests and scans that show the cancer stage. They should have had a certain kind of bladder surgery and three to six rounds of cancer drugs before. They cannot join if their cancer has spread too much, if they had cancer in other places recently, or if they got certain other treatments before. People with weak immune systems or other major health problems also cannot take part. They must agree not to become pregnant and must sign a paper saying they understand the study.<br>Study Plan Design Details:<br>This is a phase two study, which means doctors are testing if the treatment works well and is safe. There is only one group in the study. Everyone gets the same treatment. There is no placebo, and no one is kept from knowing what treatment they get. The main goal is to see if this mix of treatments can keep the bladder safe for three years. The doctors will check on patients often, up to five years after the treatment ends.<br>Arms and Interventions:<br>Everyone in the study will get two main treatments. First, they will get pembrolizumab, the immune drug, through a drip into their vein once every three weeks. They will also get radiation five days each week, for up to four weeks. Before starting, they will have a bladder test called TURBT, where a small piece of tissue is taken. During the study, they will also have scans like CT, MRI, or PET, and give samples of urine and blood. All of this helps the doctors see how well the treatment is working.<br>Primary Outcome Measures:<br>The main goal is to find out how many people can keep their bladder working without the cancer coming back or spreading for at least three years. Doctors will use special ways to measure this and see if the treatment worked as hoped.<br>Secondary Outcome Measures:<br>Doctors also want to know how long the cancer

						stays away in the bladder area, how long people live without the cancer spreading to other places, and how long people live in total. They will also count how many people still need bladder surgery later. They will watch for side effects and how bad they are. Patients will also share how they feel, like if they have pain or trouble going to the bathroom. This will help doctors understand how the treatment affects daily life.
Kidney	1	NCT07181720	Safety and Efficacy of CD70-Targeted CAR-T Therapy in CD70-Positive Advanced Solid Tumors: A Clinical Trial	https://clinicaltrials.gov/study/NCT07181720	This study is a single-arm, open-label, dose-escalating + dose-expansion clinical study, aiming to evaluate the safety and efficacy of CD70-targeted CAR-T cell preparations, and to preliminarily observe the study drug in CD70-positive advanced malignant tumors. The pharmacokinetic characteristics of CAR-T cell preparations for the treatment of patients with CD70-positive advanced malignancies were obtained and the recommended dose and infusion schedule.	Study Overview<br>This study tests a new kind of treatment called CAR-T cells, made to find and fight a marker called CD70 on cancer cells. It helps doctors check if the treatment is safe and if it works. The study looks at people with late-stage cancers like kidney, lung, thyroid, ovary, cervix, or thymus cancer. The CAR-T cells are given in three ways: into the blood, into the chest space, or into the belly. First, doctors give a low amount to check for safety. Later, they give more to learn how well it works. They also study how the treatment moves and works inside the body.<br>Eligibility Criteria<br>People in the study must be adults with solid cancers that show CD70, who have not gotten better with usual treatments like pills, surgery, or other cancer drugs. Their body must work well enough — like the heart, blood, and kidneys — and they must not have serious mental illness or infection. Women who can have babies must not be pregnant and must use safe birth control. People who had brain tumors, some virus infections, bleeding problems, or other cancers in the last few years cannot join. Also, if a person had certain heart problems or takes strong drugs like high-dose steroids, they cannot join either.<br>Study Plan Design Details<br>This is a treatment study with no fake or dummy medicine. People are not picked by chance but are placed in one of three groups, based on how the CAR-T cells are given. No one is kept blind — both doctors and patients know what they are getting. The study has two parts: first, testing small doses to

						find the best amount, and second, using that dose in more people to check safety and if it helps.<br>Arms and Interventions<br>There are three ways the CAR-T cells are given. In one group, they are given into the blood. In another group, they are placed into the chest. The last group gets them into the belly. Before getting the CAR-T cells, all people get two drugs to prepare the body: fludarabine and cyclophosphamide. These help make space for the new CAR-T cells to work better.<br>Primary Outcome Measures<br>Doctors want to find out how safe the treatment is. They will check for bad effects in the first month after getting the CAR-T cells. They will also look at how much of the treatment can be safely given.<br>Secondary Outcome Measures<br>Doctors will check how well the treatment works. They want to see if the cancer gets smaller or stops growing. They will also check how the CAR-T cells move and stay in the body over time. Doctors will look at signs of body swelling after treatment, like high levels of some blood proteins. They will measure how long people live without the cancer getting worse, and how long they live in total.
Kidney	2	NCT07175480	PET/CT-Directed Free of Therapy for Metastatic RCC Patients With IMDC Favorable or Intermediate Risk	https://clinicaltrials.gov/study/NCT07175480	This phase 2 trial aims to test the feasibility and efficiency of PET/CT-directed treatment interruption strategy in metastatic renal cell carcinoma patients with IMDC favorable/intermediate risk who achieve complete (CMR) or partial metabolic response (PMR) after ,â•12 months of first-line PD-1/PD-L1 Immune checkpoint inhibitor (ICI)+ VEGFR-tyrosine kinase inhibitor (TKI) therapy. It helps figure out whether PET/CT can safely direct treatment pause as well as explores a new individualized treatment option based on metabolic imaging for RCC patients.	Study Overview (Detailed Description and Conditions):<br>This study is testing if a break from treatment can be safely planned for people with a kind of kidney cancer called renal cell carcinoma, or RCC, that has spread. These people are in the low or middle-risk group. They must have taken a mix of two cancer drugs for at least one year. These two drugs are a type of immune therapy (called PD-1 or PD-L1 inhibitors) and a drug that blocks tumor blood supply (called VEGFR-TKI). After one year, if their cancer shrinks or is gone based on a special scan called PET/CT, they stop taking the drugs. PET/CT is a kind of scan that shows how active cancer cells are, using a sugar-like substance. The study checks if it's safe to stop the drugs and only restart them if the cancer shows signs of coming back. This may help cut down side effects, cost, and over-treatment. During the break, patients are checked often with

scans to make sure the cancer does not grow. If the scan shows the cancer is growing or coming back, treatment starts again.

Eligibility Criteria (Inclusion and Exclusion Criteria):

To join the first part, people must be grown-ups with advanced or spread kidney cancer and be in fair or good health. Their lab tests must show their organs are working well. They must agree to use birth control and not be pregnant. People cannot join if they have a type of very fast-growing tumor, had past treatment for advanced RCC, or have other bad health problems like serious heart, brain, or liver conditions. In the second part, they must have taken the drug mix for at least one year without major side effects and show that their cancer has shrunk or disappeared on the PET/CT scan. They must still be in good health and not have new cancer spots. If someone does not meet these rules or if they get worse, have strong side effects, or choose to quit, they will be taken out of the study.

Study Plan Design Details:

This is a single-arm trial, meaning all the people in the study get the same plan. Everyone starts by taking the usual two-drug mix. If the cancer responds well, they stop taking the drugs and are watched closely. The main goal is to treat people only when needed, based on the cancer activity on the PET/CT scans. No one in the study gets fake drugs or placebo. It is not hidden who gets the drugs, so both doctors and patients know what is given.

Arms and Interventions:

Everyone in the study first takes a mix of two drugs: an immune checkpoint blocker (like PD-1 or PD-L1 inhibitors) and a drug that blocks blood supply to tumors (called VEGFR-TKI). If the PET/CT scan after a year shows the cancer is gone or much smaller, the drugs are stopped. The treatment can be restarted later if the cancer comes back. The drugs used are ones already approved and sold in the market.

Primary Outcome Measures:

The main thing this study looks at is how many

						people are still doing well and without cancer growth two years after starting the treatment. This is checked by a PET/CT scan using a system called PERCIST 1.0, which looks at how active the cancer cells are.<br>Secondary Outcome Measures:<br>The study also looks at how long the cancer stays away after stopping treatment, and how many people respond if the drugs are started again. It checks how long people live, whether they have side effects, and how the treatment affects their daily life and feelings. Tools like FKSI-19, EORTC QLQ-C30, EQ-5D-5L, and HADS are used to ask about symptoms, health, and mood. It also looks at cost and time without bad side effects. They will keep samples of blood and tissue to learn more in the future.
Kidney	3	NCT07172386	Preoperative Therapy of Super-selective Tumor Artery Embolization Combined With Toripalimab and Axitinib in Advanced RCC	https://clinicaltrials.gov/study/NCT07172386	This is a phase II study to determine the efficacy and safety of Super-selective tumor artery embolization combined with toripalimab and axitinib as treatment for patients with the advanced kidney cancer . Further evaluate whether the treatment plan is beneficial to the patient's operation. Patients will undergo super-selective embolization of the feeding arteries to the renal tumour one week prior to drug therapy, followed by toripalimab administered every three weeks for three to four consecutive cycles combined with axitinib administered for four consecutive cycles in the preoperative and patients need to continue taking the drug for a year after surgery	Study Overview (Detailed Description and Conditions)<br>This study looks at a new way to treat people with a serious kind of kidney cancer before surgery. The plan uses a mix of three steps. First, the doctors block the blood flow to the tumor in the kidney. This is called “super-selective embolization,” where only the small blood pipes that feed the tumor are blocked. A week later, patients get two drugs: toripalimab and axitinib. Toripalimab helps the body’s defense system fight cancer, and axitinib blocks new blood pipes from feeding the tumor. People get these drugs before surgery in several short rounds. After the surgery, they keep taking the drugs for about a year. The goal is to see if this treatment makes the surgery safer and more helpful for the patient. This study is for people with advanced kidney cancer, also called renal cell carcinoma .<br>Eligibility Criteria (Inclusion and Exclusion)<br>People can join this study if they are adults with a confirmed type of kidney cancer and are healthy enough to go through surgery. They must not have signs that the cancer spread to the brain. Their blood, liver, and other body parts must work well. They must also be willing to avoid pregnancy

during and after treatment by using approved birth control.

People cannot join if they had past treatments like chemo, radiation, or strong immune drugs. They are also excluded if they have other cancers, strong allergies to drug parts, or certain body or immune system diseases. Heart, lung, or bleeding problems also prevent people from joining. Anyone with an ongoing infection, mental health trouble, or who is unable to afford the treatment checks, is also not allowed to join .

Study Plan Design Details

This study is a treatment trial. All patients will get the same care. There are no fake treatments or hidden groupings. Everyone knows what is being given. The goal is to test if the new plan helps and is safe for people with advanced kidney cancer .

Arms and Interventions

All patients will get the same plan. First, doctors block the blood to the kidney tumor. One week later, they start getting toripalimab every third week and axitinib at the same time. This happens for about four rounds before surgery. After surgery, patients keep getting toripalimab for about seventeen rounds. The drugs help the body fight cancer and stop new blood lines from helping the tumor .

Primary Outcome Measures

The main thing this study checks is how many people get their tumor fully removed during surgery, with no cancer left at the edge of the removed tissue. This shows if the surgery worked well. Doctors also look at how well the tumor shrinks before surgery using scan rules called RECIST .

Secondary Outcome Measures

Doctors will also look at side effects from the drugs before surgery. They will check blood loss during surgery, hospital stay, and how strong the patients feel after surgery. They will track how long it takes for the cancer to come back. They will check if the tumor has fewer cancer cells after treatment. They will ask patients how they feel during and after the

						treatment. Finally, they will see how long people live and if some body signs can help guess who benefits the most from this care .
Kidney	4	NCT07172802	A Study of GI-108, an Anti-CD73-IgG4 Fc-IL-2v Bispecific Fusion Protein, as Monotherapy in Patients With Advanced or Metastatic Solid Tumors	https://clinicaltrials.gov/study/NCT07172802	The purpose of this study is to evaluate the safety, tolerability, pharmacokinetics, and therapeutic activity of GI-108, as a single agent, in patients with advanced or metastatic solid tumors	Study Overview<br>This study is testing a new drug called GI-108 in people with serious cancers that have spread to other parts of the body. These cancers include lung cancer, cancer in the head or neck, pancreas cancer, and kidney cancer. GI-108 is a lab-made protein that can help the body's immune system fight cancer. This drug works in two ways: it blocks a protein called CD73, and it also includes a changed form of a natural body protein called Interleukin-2, which helps white blood cells grow. This study will look at how safe the drug is, how it moves through the body, and how well it works to slow down or shrink the cancer. The study has two parts. In the first part, small groups of people will get different amounts of the drug to find out the best safe dose. In the second part, more people will get that dose to see how well it works.<br>Eligibility Criteria<br>People can join this study if they are adults and have strong enough body systems like liver and kidney to handle the treatment. They must have cancer that can be measured through scans, be able to walk and take care of themselves, and not be very sick from past treatments. If they have HIV, the virus must be under control with medicine. People cannot join if their cancer has spread to their brain, or if they have other active cancers or certain infections like hepatitis or tuberculosis. People with serious liver problems, certain immune system diseases, or those taking strong steroids cannot join either.<br>Study Plan Design Details<br>This is a treatment study. People will be placed into groups in a set order, not randomly. Everyone will know what they are getting because there is no pretend medicine used. First, small groups will get different amounts of GI-108 to see what amount is safe. Later, more people will get the best safe dose. The main goal is to see if the drug is safe and helps

						treat the cancer.<br>Arms and Interventions<br>Everyone in the study will get the GI-108 drug. It will be given through a vein once every three weeks. At first, people will get small doses, and the amount will go up slowly to find a safe level. Then, more people will get the dose that works best. The drug will be given for up to two years, as long as it is helping and there are no bad side effects.<br>Primary Outcome Measures<br>The study will first look at how many people get strong side effects that stop them from getting more doses. It will also look at how many people have side effects caused by the immune system, which fights infections but sometimes attacks the body. In the second part of the study, it will check if tumors get smaller or stay the same when scanned by doctors.<br>Secondary Outcome Measures<br>Other things the study will check include how long the cancer stays the same or gets smaller, how long people live without the cancer getting worse, and how long they live overall. The study will also look at how the drug spreads in the body, how long it stays, and how fast it leaves the body. It will measure how the immune system changes and whether the body makes proteins that might block the drug.
Kidney	5	NCT07167329	Real-World Effectiveness and Pharmacogenetics of Belzutifan in VHL Syndrome: The BELIEVE-VHL Trial	https://clinicaltrials.gov/study/NCT07167329	The BELIEVE-VHL Trial is a prospective real-life study designed to evaluate the therapeutic effects, benefits, and adverse effects of belzutifan, as well as the timing of treatment response and disease progression in patients with von Hippel-Lindau (VHL) syndrome.	Study Overview<br>This study wants to learn how well a drug called belzutifan works for people who have a rare illness called von Hippel-Lindau syndrome. This illness causes tumors in many parts of the body like the brain, eyes, kidneys, and other places. The drug may help shrink these tumors or slow them down. The study also checks how the drug affects the body, when it starts to work, and what side effects happen. The study is being done in Brazil with people who have this illness, and the drug will be given by mouth once a day. The study also looks at the cost of care when using this drug compared to not using it.<br>Eligibility Criteria

To join the study, a person must be at least in their early teens. They must have a clear test result that shows they have von Hippel-Lindau syndrome. They also need to have tumors that can be seen in scans or are growing. The person must feel well enough to do daily things and be able to take pills by mouth. People who do not have this illness, are very sick with heart problems, have another kind of cancer, or infections like HIV or hepatitis cannot join. Also, anyone who had surgery or radiation too close to the start of the study or cannot stick to taking the drug every day will not be able to join.

Study Plan Design Details

This is a treatment study. Everyone in the study gets the same drug, belzutifan. The study is open-label, which means both the doctors and the patients know what drug is being given. This drug will be taken by mouth once each day. The study will follow each person to see how their tumors change and if they feel better or worse. People age fourteen or older can join, no matter how far along their illness is. The study includes many kinds of tumors linked to von Hippel-Lindau syndrome.

Arms and Interventions

Everyone in the study will take the same drug. People with von Hippel-Lindau syndrome who have tumors that need care will take a pill called belzutifan, which is also known by the name Welireg. This drug works by blocking something in the body that helps tumors grow. The drug is given by mouth once a day.

Primary Outcome Measures

The main thing this study looks at is how much the tumors shrink or stop growing after people start the drug. This is done by taking scans of the body at the start, then after about three months, six months, a year, and later. Different kinds of scans are used depending on where the tumors are. The study checks how many people have smaller tumors or tumors that stay the same during this time.

Secondary Outcome Measures

The study also checks if the drug causes a drop in red blood cells, which is called anemia. They check

						blood levels over time to see if they change. Some people may need extra medicine or even a blood transfusion if their red blood cell levels get too low. The study also asks people how they feel during the treatment using a survey that looks at pain, mood, and daily life. It also checks if the cost of care goes up or down when people take the drug. This includes costs from doctor visits, hospital stays, and time off work. These checks go on for about two years.
Testis	1	NCT07118176	Determining the Biodistribution of an Imaging Tracer (68Ga-FAPi-46) in Patients With Solid Tumors or Hematologic Cancers	https://clinicaltrials.gov/study/NCT07118176	This phase I trial is evaluating a new imaging tracer (68Ga-FAPi-46) with positron emission tomography (PET)/computed tomography (CT) to determine where and to which degree the tracer (68Ga-FAPi-46) accumulates in normal and cancer tissues (the biodistribution) in patients with solid tumors or hematologic (blood) cancers. PET is an established imaging technique that utilizes small amounts of radioactivity attached to very minimal amounts of tracer, in the case of this research, 68Ga-FAPi-46. Because some cancers take up 68Ga-FAPi-46, it can be seen with PET. CT utilizes x-rays that traverse the body from the outside. CT images provide an exact outline of organs and potential inflammatory tissue where it occurs in a patient's body. Combining a PET scan with a CT scan can help make the image easier to interpret. PET/CT scans are hybrid scanners that combine both modalities into a single scan during the same examination.	Study Overview<br>This study looks at how a type of surgery called retroperitoneal lymph node dissection, or RPLND, affects how men pee and their sexual health after testicular cancer. This surgery removes lymph nodes in the belly and is used when other treatments, like chemo, do not work or are not an option. Doctors already know that this surgery can make it harder for men to ejaculate. But they are not sure how it affects other things, like urine flow or getting and keeping an erection.<br>To learn more, doctors will follow adult men who are having this surgery. They will ask the men to answer questions about their pee habits, ability to have sex, and life quality. These questions will be asked before and after surgery, up to about half a year later. Men will also take simple tests, like peeing into a special machine that checks the strength of urine flow.<br>This will help doctors better understand all the changes that might happen after surgery. It will also help them explain things more clearly to future patients and offer better care afterward.<br>Eligibility Criteria<br>Only adult men with testicular cancer who are getting this belly lymph node surgery can join the study. Men who already had this surgery before or who do not give their permission cannot take part. Healthy men who do not have cancer are not part of this study.<br>Study Plan Design Details<br>This is an observational study. That means doctors are not giving new treatments. They are just

						watching and asking questions over time. The men will all be in the same group and followed from before surgery to about half a year later. Doctors want to see how their body and health change during this time. Arms and Interventions There is one group in this study. It includes adult men with testicular cancer who are getting RPLND surgery. Each man will take simple tests and fill out forms before and after surgery. These forms ask about peeing, erections, and ejaculation. The pee flow test checks how fast and well urine comes out. The forms used are called IPSS, ICIQ-MLUTS, IIEF-5, MSHQ-EjD, and EQ-5D-5L, which are standard forms to understand how someone feels about their pee and sex life. Primary Outcome Measures The main thing doctors want to learn is how the surgery changes the way urine flows. They will check this using a test called uroflowmetry before and after surgery. They will also check how much urine stays in the bladder after peeing, which can show if the bladder is emptying well. Secondary Outcome Measures Other things the doctors want to find out are how the surgery affects erection, ejaculation, and general comfort when peeing. Men will answer short forms that ask how easy or hard it is to get an erection or ejaculate. These will be asked before surgery and again for up to six months later. Doctors will compare the answers to see how much things change over time.
Testis	2	NCT06954233	Voiding and Erectile Function After Retroperitoneal Lymph Node Dissection for Testicular Cancer	https://clinicaltrials.gov/study/NCT06954233	"This study aims to evaluate how retroperitoneal lymph node dissection (RPLND), a surgical treatment for testicular cancer, may affect urinary and sexual functions in men. RPLND involves the removal of lymph nodes from the abdominal area and is sometimes necessary in patients who are not eligible for chemotherapy or who have residual disease after chemotherapy. While this surgery is known to carry a risk of affecting ejaculation, its potential impact on other areas such as urination or erection is not well understood.	Study Overview (Detailed Description and Conditions): Testicular cancer is not common, but it is the top solid cancer in young men. Most of these cancers start from cells that grow into sperm, called germ cell tumors. These tumors come in two types: seminoma and non-seminoma. One out of five men with seminoma have cancer that spreads to belly lymph nodes but not beyond. Doctors often treat these men with strong drugs or beams of radiation. These work well but can cause harm later, such as

The study will prospectively follow adult men undergoing RPLND. It will assess changes in lower urinary tract symptoms, urine flow, ejaculation, erection, and overall quality of life before surgery and during follow-up visits up to 6 months after the operation. Patients will complete standardized questionnaires and undergo simple, non-invasive tests such as urine flow measurement.

By identifying how RPLND may influence urinary and sexual health, this study seeks to improve understanding of the full range of effects of this treatment. The findings may help clinicians better inform patients before surgery and support improved post-operative care."

heart problems or other cancers. Some men may lose their hearing, feel numbness, or have trouble breathing. It can also lower their ability to have kids. This study looks at a different treatment called retroperitoneal lymph node dissection, or RPLND. This is surgery to take out lymph nodes from the back of the belly. The goal is to see if this surgery alone can help without the side effects of drugs or radiation.

Eligibility Criteria (Inclusion Criteria and Exclusion Criteria):

Men can join if they are adults and had testicular seminoma with no signs of other types of tumors. They must have had one or two small swollen lymph nodes in the back of the belly. These nodes must be under a certain size and close to the testicle. Their blood tests must be normal before surgery. Men cannot join if they had other tumor types or if the surgeon thinks surgery is not safe. Also, if one tumor marker called AFP was too high before or after testicle removal, they cannot join.

Study Plan Design Details:

This study is in its second phase. That means it is still learning how well the treatment works and how safe it is. Everyone in the study gets the same treatment. There is no fake or test group. The study is open, so both doctors and patients know what treatment is given. The main reason for the study is to treat cancer and check if it comes back.

Arms and Interventions:

All patients in the study had seminoma that came back or was found to be in an early stage that already spread to a few lymph nodes. They will all have the same surgery. This is called retroperitoneal lymph node dissection. It removes lymph nodes on both sides of the belly while saving nearby nerves.

Primary Outcome Measures:

The main goal is to check if the cancer stays away for two years. They will look at scans and blood tests. If there are no signs of cancer and tests stay normal, the treatment worked.

Secondary Outcome Measures:

The study will also look at what side effects happen

						after surgery. They will check how long each person stays in the hospital. They will see how many people need no other treatment like drugs or radiation after surgery. They will count how many stay alive without cancer for two years and how many are still alive for any reason in that time.
Testis	3	NCT06932458	A Clinical Trial of Primary Retroperitoneal Lymph Node Dissection in Patients With Testicular Seminoma With Limited Retroperitoneal Metastases	https://clinicaltrials.gov/study/NCT06932458	"Testicular cancer represents 1% of adult neoplasms and is the most common solid malignancy in young men. At diagnosis, approximately 90% of cases are germ cell tumours (GCT), categorised as either seminoma (55-60%) or non-seminoma types (40-45%).<br><br>For many years, the management of patients with CS IIA/B seminoma and retroperitoneal lymph node involvement ‚â§ 3 cm are eligible for treatment with either radiotherapy or chemotherapy Despite high cure rates for CS II seminoma (approximately 90%) with chemotherapy or radiotherapy, concerns persist regarding short and long-term treatment-related toxicities (such as increased risks of cardiovascular disease and secondary malignancies As such, an alternative strategy which has been explored in this study is the role of RPLND for the management of these patients"	Study Overview<br>This study checks if a small daily dose of aspirin can help stop blood clots in people with a type of cancer called germ cell tumor. These tumors often happen in the testicles. People in this study also get usual cancer drugs, including one called cisplatin. Blood clots are a serious problem that can happen during cancer treatment. The study will look at how many people stay free of clots for six months. It will also see if taking aspirin is safe. The study will take place at a few cancer centers in the United States.<br>Eligibility Criteria<br>People can join if they are adults under 70 years old and have a certain stage of germ cell or testicular cancer. They must be planning to get usual cancer treatment with certain chemo drugs. They must also be at a high risk of clots based on cancer stage, blood test results, or other health risks. People cannot join if they already take blood-thinning drugs, had a clot before, have a bleeding problem, or are allergic to aspirin.<br>Study Plan Design Details<br>The study will not compare two groups. Everyone in the study will get the same treatment. This is called a single-group design. There will be no fake treatment or sugar pills. The goal is to stop clots before they happen. The study is in Phase Two, which means it checks how well and how safe the treatment is. The study will start with a small group, and if the treatment looks safe and helpful, more people will join.<br>Arms and Interventions<br>Everyone in the study will take low-dose aspirin, which is a drug also called acetylsalicylic acid. They will take one small pill by mouth each day for about six months. If a person gets a blood clot, they will stop taking aspirin and start a different treatment to

						thin the blood.<br>Primary Outcome Measures<br>The main thing the study will look at is whether people stay free from blood clots for six months after starting chemo. This includes clots in the legs, lungs, or other parts of the body. It also counts if a person dies because of a clot.<br>Secondary Outcome Measures<br>The study will also look at other things like if a person bleeds too much, if they bleed in a way that needs care, or if they feel pain from the bleeding. It will check if the cancer comes back or if the person passes away for any reason. The study will also track a rare side effect called febrile neutropenia, which means a low count of white blood cells with a fever, to see if it might be caused by aspirin.
Testis	4	NCT06866964	A Single-arm, Phase II Clinical Trial of ASPIRin to prEvent Venous Thromboembolism in Patients With Advanced Germ Cell Tumors Receiving Chemotherapy	https://clinicaltrials.gov/study/NCT06866964	The purpose of this study is to the 6-month Venous Thromboembolism (VTE)-free rate in participants with advanced germ cell cancer at high risk of VTE who are receiving standard of care cisplatin-based chemotherapy and low-dose acetylsalicylic acid (ASA) and compare to relevant historical controls	Study Overview:<br>This study looks at two ways to remove a testicle when doctors think there might be testicular cancer. One way is the usual surgery, where the outer stomach muscle layer is cut. The other way tries not to cut that muscle. The study will check if the second way causes less pain, uses fewer pain pills, or has fewer nerve problems. Both surgeries take out the testicle and the cord that holds it, but the newer way might hurt the nerve less and help people heal faster. Doctors are not sure yet if one way is better, so this study will compare them.<br>Eligibility Criteria:<br>Men who are at least eighteen years old and are having this surgery because the doctor thinks they may have testicle cancer can join. They must not already use strong pain pills for other problems. Even men with cancer that has spread can join if they don't need pain pills for that. Men who had drugs, like opioids or other street drugs (not weed), or who had radiation or chemotherapy before surgery, cannot join. Men needing a larger cut than usual also cannot join.<br>Study Plan Design Details:<br>This is a test where people are split into two groups by chance. Only the person getting surgery will not know which kind they get. The goal is to see which

						method helps with healing and pain after surgery.<br>Arms and Interventions:<br>One group will have the usual surgery where the muscle layer is cut. The other group will have surgery that tries to leave that muscle alone. Both groups will still have their testicle and cord taken out the same way.<br>Primary Outcome Measures:<br>Doctors will ask men how much pain they feel in the first week after surgery. They will use a score that goes from no pain to very bad pain.<br>Secondary Outcome Measures:<br>Men will tell doctors how many pain pills they take that first week. They will also be asked about any nerve pain they feel. Nerve pain means a feeling of tingling, numbness, or burning in the area. The doctors will check if there are any problems from the surgery like bad healing or other issues in that first week or at a follow-up visit.
Testis	5	NCT06828185	Patient Reported Experiences With Sparing External Oblique Fascia Vs Standard Inguinal Orchiectomy	https://clinicaltrials.gov/study/NCT06828185	"The purpose of this study is to evaluate the difference in patient-reported postoperative outcomes between two standard-of-care surgical techniques for radical orchiectomy (inguinal orchiectomy versus external oblique fascia sparing orchiectomy) for treatment of patients with suspected testicular malignancy. The main questions it aims to answer are:<br><br>1. Does sparing the external oblique fascia during orchiectomy reduce pain after surgery?<br>2. Is there a difference in narcotic consumption after surgery?<br>3. Is there a difference in neuropathic pain after surgery?<br>4. Is there a difference in complications after surgery?"	Study Overview (Detailed Description and Conditions):<br>This study is testing if a new drug called tarlatamab is safe when used with radiation. The drug is meant to help the body's immune system fight tumors that show a certain marker called DLL3. The study will first treat tumors outside the brain. If that goes well, the study may try to treat brain tumors next. If giving the drug and radiation at the same time causes problems, the study will try giving them one after the other. If even that is not safe, then patients will only get the drug. The study will also look at how well the drug and radiation work to shrink tumors. These tumors are from types like skin cancer, thyroid cancer, brain cancer, bladder cancer, testicular cancer, lung cancer, and others. All these cancers are known to often have DLL3. Up to thirty people will be in the study.<br>Eligibility Criteria (Inclusion and Exclusion Criteria):<br>To join this study, people must be at least eighteen years old. They need to have a tumor that came back or did not get better with other treatments. The tumor must be one of the types that often show

DLL3. People also must be healthy enough to handle the drug and radiation. They need to have at least one tumor that can be treated with radiation. People cannot join if they have had certain serious health issues, like bad heart problems or serious infections. They also cannot join if they had bad side effects from similar treatments before. Women who are pregnant or planning to be cannot join. Men and women must agree to use birth control. People who cannot follow the study rules will not be allowed to take part.

Study Plan Design Details:

This study is not a random test. All patients know what they are getting. The first group will get the drug and radiation at the same time for tumors outside the brain. If that is safe, the next group will get both treatments for brain tumors. If it is not safe, another group will get the drug and radiation one after the other. If that is also not safe, the last group will only get the drug. The main goal is to check safety first, and then see how well it works.

Arms and Interventions:

The study has four groups. One group gets the drug with radiation to tumors outside the brain. Another group gets the drug with radiation to brain tumors. A third group gets radiation first or after, with the drug given at a different time. The last group only gets the drug. The drug is given by vein in a step-up dose, starting low and then increasing. The radiation is given as needed depending on which group the patient is in.

Primary Outcome Measures:

The main thing this study wants to find out is how safe the drug and radiation are together. They will look for serious side effects in the first eight weeks after radiation starts. If someone has strong bad effects or has to stop the drug early, that counts as not safe. The study will count how many people this happens to.

Secondary Outcome Measures:

The study will also check how well the tumors respond to the treatment. They will see if the tumor gets smaller or goes away for a while. They will

	count how many people have this happen. They will also check how long people live and how long they stay free from cancer getting worse. They will watch for side effects and compare them in people with different types of tumors. If a person’s tumor shrinks, they will see how long that lasts.

Table S2. Definitions for Independent Assessment from ClinicalTrials.gov Submission Process.

Title	Data Element Definition
**Brief Summary**	Definition: A short description of the clinical study, including a brief statement of the clinical study’s hypothesis, written in language intended for the lay public.<br>More info and guidelines at: https://clinicaltrials.gov/submit-studies/prs-help/plain-language-guide-write-brief-summary
**Conditions or Focus of Study**	Definition: The name(s) of the disease(s) or condition(s) studied in the clinical study, or the focus of the clinical study. Use, if available, appropriate descriptors from NLM’s Medical Subject Headings (MeSH)–controlled vocabulary thesaurus or terms from another vocabulary (e.g., SNOMED CT) that has been mapped to MeSH within the Unified Medical Language System (UMLS) Metathesaurus.
**Eligibility Criteria**	Definition: A limited list of criteria for selection of participants in the clinical study, provided in terms of inclusion and exclusion criteria and suitable for assisting potential participants in identifying clinical studies of interest. Use a bulleted list for each criterion below the headers "Inclusion Criteria" and "Exclusion Criteria".
**Interventional Study Model**	Definition: The strategy for assigning interventions to participants.<br>- Single Group: Clinical trials with a single arm.<br>- Parallel: Participants are assigned to one of two or more groups in parallel for the duration of the study.<br>- Crossover: Participants receive one of two (or more) interventions during the initial phase of the study and then receive the other intervention during the second phase.<br>- Factorial: Two or more interventions, alone or in combination, are evaluated in parallel against a control group.<br>- Sequential: Groups of participants are assigned to receive interventions based on milestones (e.g., dose escalation, adaptive design).<br>Model Description: Provide details about the Interventional Study Model.
**Arms**	Definition: A description of each arm of the clinical trial that indicates its role in the trial, provides an informative title, and, if necessary, additional descriptive information (including interventions administered in each arm) to differentiate each arm.<br>Note: "Arm" means a pre-specified group or subgroup of participants in a clinical trial assigned to receive specific intervention(s) (or no intervention) according to a protocol.
**Interventions**	Definition: Specify the intervention(s) associated with each arm or group; at least one intervention must be specified for interventional studies. For observational studies, specify the intervention(s)/exposure(s) of interest, if any. If the same intervention is associated with more than one arm or group, provide the information once and use the Arm or Group/Intervention Cross-Reference to associate it with more than one arm or group.

**Primary Outcome Measure**	Definition: A description of each primary outcome measure (or, for observational studies, specific key measurement[s] or observation[s]) used to describe disease patterns, traits, or associations with exposures, risk factors, or treatment.<br>Note: "Primary outcome measure" means the outcome measure(s) of greatest importance specified in the protocol, usually the one(s) used in power calculation. Most studies have one primary outcome measure, but more than one may be allowed.
**Secondary Outcome Measure**	Definition: A description of each secondary outcome measure (or, for observational studies, specific secondary measurement[s] or observation[s]) used to describe disease patterns, traits, or associations with exposures, risk factors, or treatment.<br>Note: "Secondary outcome measure" means an outcome measure of lesser importance than a primary one, but still part of a pre-specified analysis plan to evaluate effects of interventions or exposures. Clinical studies may have more than one.
